# Vocabulary relearning in semantic dementia: Positive and negative consequences of increasing variability in the learning experience

**DOI:** 10.1016/j.neuropsychologia.2015.01.015

**Published:** 2015-09

**Authors:** Paul Hoffman, Natasha Clarke, Roy W. Jones, Krist A. Noonan

**Affiliations:** a Neuroscience and Aphasia Research Unit (NARU), University of Manchester, UK; b Centre for Cognitive Ageing and Cognitive Epidemiology (CCACE), Department of Psychology, University of Edinburgh, 7 George Square, Edinburgh EH8 9JZ, UK; c Research Institute for the Care of Older People (RICE), Bath, UK

**Keywords:** Semantic dementia, Anomia therapy, Word relearning, Generalisation, Conceptual knowledge

## Abstract

Anomia therapy typically aims to improve patients' communication ability through targeted practice in naming a set of particular items. For such interventions to be of maximum benefit, the use of trained (or relearned) vocabulary must generalise from the therapy setting into novel situations. We investigated relearning in three patients with semantic dementia, a condition that has been associated with poor generalisation of relearned vocabulary. We tested two manipulations designed to improve generalisation of relearned words by introducing greater variation into the learning experience. In the first study, we found that trained items were retained more successfully when they were presented in a variety of different sequences during learning. In the second study, we found that training items using a range of different pictured exemplars improved the patients' ability to generalise words to novel instances of the same object. However, in one patient this came at the cost of inappropriate over-generalisations, in which trained words were incorrectly used to name semantically or visually similar objects. We propose that more variable learning experiences benefit patients because they shift responsibility for learning away from the inflexible hippocampal learning system and towards the semantic system. The success of this approach therefore depends critically on the integrity of the semantic representations of the items being trained. Patients with naming impairments in the context of relatively mild comprehension deficits are most likely to benefit from this approach, while avoiding the negative consequences of over-generalisation.

## Highlights

• SD patients attempted to recover lost vocabulary by naming pictures daily.
• Learning was more successful when pictures presented in a different order each day.
• One patient benefitted from naming multipleexemplars of each item.
• But in the other patient, this resulted in over-generalisation of the trained name.

## 1 Introduction

Word-finding difficulty (anomia) is a key presenting symptom in almost all forms of aphasia and is the most commonly addressed ability in impairment-based aphasia therapies (Nickels, 2002). The goal of anomia therapies is always the same: to improve the patient's word retrieval ability, thereby increasing the expressive vocabulary available to them in everyday situations. The success of this approach therefore depends critically on the patient's ability to generalise gains made in the training setting to novel situations. Many studies have assessed the degree to which training on a particular set of words results in improvement for other words that were not included in the therapeutic intervention. The evidence suggests that this form of generalisation is typically very limited (Croot et al., 2009; Nickels, 2002). In this study, we use the term generalisation in a slightly different way. We were interested in how successfully patients are able to generalise knowledge for the items treated in therapy when they encounter those same items in novel settings. This could include apparently trivial changes to the setting, such as presenting the stimuli used in therapy in a different order to the one the patient experienced during therapy sessions, or it might include more major changes to the therapy stimuli themselves.

Generalisation of this form has received much less attention but is critical for ensuring that interventions have maximum benefit for patients in everyday situations and not only within the narrow confines of the therapy setting (Croot et al., 2009; Herbert et al., 2003). Anomia therapies often use a single picture as a naming cue for a particular word and assume that naming of this one stimulus will generalise to the diverse, and often visually dissimilar, range of other examples of the same object that could be encountered in the world (see Fig. 6 for examples). In addition, most anomia therapies feature highly specific tasks (repetition, cued naming and so on) which are focused around a limited pool of items and administered in a relatively rigid or fixed order. The goal of the present study was to explore whether increasing variability within the learning experience would improve the success of anomia therapy by promoting generalisation. We investigated this using a series of interventions in three patients with semantic dementia (SD). Although SD is a relatively rare disorder and is not typical of all forms of aphasia, the advantage of using this population is that generalisation is known to be a particular weakness in this group and has received some attention in the rehabilitation literature (Graham et al., 1999; Heredia et al., 2009; Mayberry et al., 2011a; Snowden and Neary, 2002).

SD (also known as the semantic variant of primary progressive aphasia) is a neurodegenerative condition whose primary presenting symptom is a progressive loss of semantic knowledge (Gorno-Tempini et al., 2011; Hodges and Patterson, 2007). The degradation of semantic knowledge, which is associated with atrophy to anterior temporal cortex (Butler et al., 2009; Nestor et al., 2006), is multi-modal, affecting comprehension of words as well as object use and recognition of objects from vision, sound, taste and smell (Bozeat et al., 2000, 2002; Luzzi et al., 2007; Piwnica-Worms et al., 2010). In most patients, however, the most prominent symptom is a pronounced anomia. Patients experience a progressive reduction in expressive vocabulary, with general, superordinate terms like “thing” and “place” and “do” increasingly replacing more specific terms in speech (Bird et al., 2000; Hoffman et al., 2014). It is important to note, however, that anomia in SD is less a word-finding difficulty and more a word-*knowing* difficulty. In other words, the patients' anomia appears to be a direct consequence of the degradation of the underlying semantic knowledge store, as evidenced by the increasingly non-specific responses given in picture naming (e.g., swan→duck→bird→animal; Hodges et al., 1995) and the strong relationship between naming ability and the familiarity and typicality of the concepts being probed (Hoffman et al., 2012; Woollams et al., 2008).

A number of studies have used naming therapies to treat anomia in SD patients (e.g., Bier et al., 2009; Henry et al., 2013; Heredia et al., 2009; Jokel et al., 2006, 2010; Mayberry et al., 2011a; Savage et al., 2013; Snowden and Neary, 2002). Given that anomia in these patients is related to loss of knowledge of word meaning, this process is often referred to as “word relearning”. Most studies have found that repeated practice in naming pictures can lead to substantial improvements in naming for those items, albeit with a number of important caveats. The first is that the success of relearning is dependent on the degree of residual semantic knowledge for the trained items, with therapy gains most likely for items that patients still recognise and demonstrate understanding of Jokel et al. (2006, 2010) and Snowden and Neary (2002). The second is that trained knowledge often fades quickly once regular practice stops (Croot et al., 2009; Graham et al., 1999; Savage et al., 2013). The third is that generalisation of the trained words to novel situations is limited. Snowden and Neary (2002), for example, conducted a study in which a patient with SD learned to name 20 pictures through repeated practice over a period of three weeks. Following this training, the patient was able to name all of the pictures correctly. However, her performance deteriorated substantially when the pictures were presented in a different context to that used during training (on different sheets of paper and in a different sequence). Another well-known case is that of patient DM (Graham et al., 1999), who spent long periods practicing recall of lists of words from different categories. He typically recalled items in exactly the same order as they appeared in the training materials, suggesting that he relied mainly on “rote learning” to memorise the items. Other studies have found that patients show limited generalisation to novel exemplars of the objects used during training, particularly if these are visually dissimilar (Heredia et al., 2009; Mayberry et al., 2011a).

A number of researchers have interpreted these findings within the complementary learning systems theory of knowledge acquisition (Graham et al., 1999; Heredia et al., 2009; Mayberry et al., 2011a; McClelland et al., 1995). This theory posits a neural division of labour between hippocampal and medial temporal lobe structures that are critically involved in initial coding of new memories and neocortical sites that are involved in representing knowledge over the longer term (see also Alvarez and Squire, 1994). This view holds that the hippocampal system is able to rapidly encode the specific details of individual learning episodes. To achieve this goal, it employs a sparse coding system in which individual experiences are clearly differentiated from one another. Over time, the details of these individual episodes are transferred to the neocortex through a process of gradual consolidation. Importantly, the consolidation process extracts statistical regularities that are true across a whole series of experiences, while discarding the idiosyncratic aspects of each individual episode. This process results in the acquisition of semantic knowledge that reflects the typical characteristics of objects and events in the world, rather than the details of individual experiences. Because knowledge in the neocortical system is no longer tied to specific experiences, it can readily be generalised to novel situations. How does this theory explain the poor generalisation demonstrated by SD patients in relearning studies? It has been claimed that, due to damage to the neocortical semantic system, patients are particularly reliant on the hippocampal system for representing information learnt during therapy. This allows the patients to learn the association between a particular picture they are exposed to during training and the word used for this picture, but the specific nature of the hippocampal trace means that they have difficulty generalising the name to new instances of the same type of object. In the same vein, when patients attempt to recall trained information they do so by recalling the specific details of the learning experience, which results in apparent “rote learning” effects in which they tend to rigidly recall items in the same order that they were encountered during training.

Over-reliance on hippocampal learning is at one level a reasonable strategy for patients with SD, in that it allows them to reliably associate pictures with names within the narrow confines of the training setting. However, it is problematic in the long term because it hampers their ability to generalise their learning to novel situations. In the present study, we tested two manipulations aimed at improving the usefulness and generalisability of names acquired during a relearning therapy programme. Classical learning theory holds that greater variability of experience during learning leads to more successful recall, particularly when learned information must be recalled in a novel context (Anderson and Bower, 1972; Smith et al., 1978). With this in mind, we designed two manipulations that increased the variability of the training experience. In the first study, we manipulated a low-level factor: the order in which items were presented during relearning. Patients practiced producing names in response to pictures of objects over a period of three weeks. In one condition, the pictures were presented in the same order each day, as is typically the case in interventions of this kind. In the other condition, they were presented in a different order each day. We found that the variation in order had a beneficial effect on learning, allowing the patients to better generalise their knowledge to a novel order at follow-up. In the second study, we investigated a factor that has a more direct bearing on the semantic deficits experienced by SD patients: generalisation of word learning to novel exemplars. We varied the learning experience by training patients to name three different exemplars of each object and contrasted this with an equivalent amount of training with only one exemplar. We found that training with multiple exemplars of the same object improved generalisation to new examples of the object. However, in one patient, this came at the cost of incorrect generalisations to other objects that were visually similar.

## 2 Study 1: effect of varying item order during training

### 2.1 Participants

Three individuals with SD took part in the study.

**Fig. 1:**
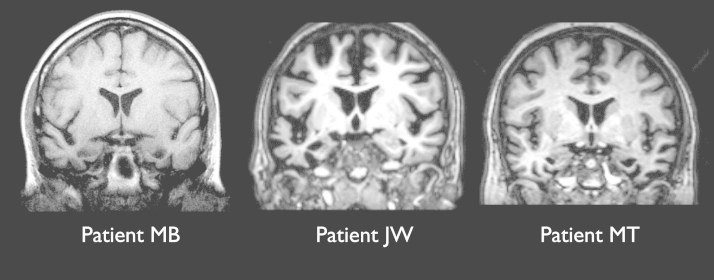
MRI scans for each patient Images are presented in neurological convention (left on left).

MB originally presented in 2004 at the age of 56, when she had been experiencing word-finding difficulties for unusual words (e.g., “matinée”, “gurkha”). Clinical examination and neuropsychological testing revealed no abnormalities at this time. However, three years later she was seen again and she now reported increasing word-finding difficulty, problems understanding words and following conversations, as well as difficulty with reading and spelling. Neuropsychological tests at this point indicated impairments in naming, verbal and non-verbal comprehension and surface dyslexia. Other aspects of cognition were preserved and she was well-oriented in time and space. An MRI scan revealed bilateral temporal lobe atrophy, particularly in the left hemisphere (see Fig. 1). A provisional diagnosis of semantic dementia was made. MB was regularly followed up over the next few years and a gradual deterioration in semantic knowledge was observed. She continued to work part-time as an auxiliary nurse for some time but retired in 2009, after finding that she was having difficulty understanding and producing the names of medical instruments. She remained an active participant in her local church group and girl guides organisation. She also participated in a number of our research studies on deficits in semantic cognition. When we discussed the possibility of her taking part in a relearning study, she was happy to take part.

MB completed a neuropsychological assessment battery in January 2012, prior to participating in Study 1, and again in March 2013, prior to Study 2. The results are shown in Table 1. The overall picture was very similar at both assessment points. She registered no general cognitive impairment on the Mini-Mental State Examination (Folstein et al., 1975) but was impaired on the Addenbrooke's Cognitive Examination-Revised (ACE-R; Mioshi et al., 2006), which includes more detailed testing of language and semantic knowledge. Semantic knowledge was assessed using components of the Cambridge Semantic Battery (Bozeat et al., 2000). MB exhibited word-finding difficulties in both picture naming and category fluency. She was also severely impaired in word-to-picture matching and on the picture version of the Camel and Cactus Test, a non-verbal test of semantic association. Verbal comprehension was assessed using a 96-item synonym judgement test (Jefferies et al., 2009) and was also impaired. In contrast, visuospatial abilities were intact, as was digit span and non-verbal reasoning, and letter fluency was only mildly impaired (as one would expect in a patient with diminished word knowledge). In short, the neuropsychological profile of MB was typical of moderate-stage SD, with multi-modal semantic deficits but no impairment to other aspects of cognition.

JW initially presented in 2010 at the age of 63. She had spent her working life as a secretary but had taken early retirement eight years previously. She had been experiencing worsening difficulties remembering the names of objects and people. The main feature of clinical examination and neuropsychological assessment at this time was a pronounced naming impairment, although verbal comprehension was also mildly impaired. In particular, she scored below the first percentile on the Graded Naming Test (Warrington, 1997). Otherwise, she was well-oriented in time and space and other aspects of cognition were entirely normal. A diagnosis of semantic dementia was made. An MRI scan carried out in 2012 indicated temporal lobe atrophy, which was markedly more severe in the left anterior temporal lobe than the right (see Fig. 1). JW became an enthusiastic participant in our research programme. She and her husband were extremely motivated to take part in any activities that might have a beneficial effect on her cognition. They were very keen to take part in word relearning studies and reported that JW already made lists of words she had forgotten and practiced these at home.

JW completed a neuropsychological assessment battery in May 2012, prior to participating in Study 1, and again in February 2013, prior to Study 2 (see Table 1). Both assessments produced a consistent picture, which was similar to that of MB. Naming and verbal and non-verbal comprehension were all impaired, while visuospatial function, working memory and executive functions remained intact. It is interesting to note that, relative to MB, JW showed a larger disparity between expressive and receptive semantic tasks. While her picture naming scores were similar to MB's, and her category fluency performance was slightly worse, she out-performed MB on tests of verbal and non-verbal comprehension. This is consistent with the left-dominant atrophy pattern observed on JW's MRI scan. Patients with predominately left temporal lobe damage often show poor naming ability, relative to comprehension, than those with more symmetrical or right-dominant damage (Lambon Ralph et al., 2001).

MT presented in 2008, at the age of 60, with a two-year history of word-finding difficulties and behavioural changes. She had difficulty in remembering the names of objects as well as recognising and naming people. Her husband also reported that she had developed unusual social behaviours, including singing at inappropriate times and kissing colleagues at work. Upon clinical assessment, she was well-oriented in time and place but exhibited mild impairments in picture naming and comprehension. Other aspects of cognition were well-preserved. MRI scan performed at this time indicated pronounced temporal lobe atrophy, which was more severe in the right hemisphere. A provisional diagnosis of semantic dementia was made, though it was noted that the unusual social behaviour was also consistent with behavioural-variant frontotemporal dementia. MT participated in our research programme over the next few years, during which time there was a gradual deterioration in her naming ability and verbal and non-verbal comprehension. Her behaviour did not change markedly during this time, though she did develop obsessive cleaning routines. She remained high-functioning in everyday life, often travelling by herself to the university for study appointments. When we approached MT to invite her to take part in the present study, her semantic knowledge had deteriorated somewhat. She was willing to take part in the study, though it is worth noting that she was the least motivated of the three patients and that her husband was sceptical about her ability to relearn vocabulary.

MT completed a neuropsychological assessment battery in September 2012, prior to participating in Study 1 (see Table 1; she did not participate in Study 2). At this stage, her naming impairment, as assessed by picture naming and category fluency, was the most severe of the three patients. Her performance on word–picture was also markedly impaired. In contrast, she was relatively more successful in making verbal synonym judgements, though this was also far below the level achieved by healthy participants. Other aspects of cognitive function were relatively spared, with the exception of the incomplete letters test of the VOSP and a mild impairment in letter fluency. However, her MMSE and ACE-R scores were the lowest of the three patients, suggesting that she had the lowest level of general cognitive function.

### 2.2 Design

**Fig. 2:**
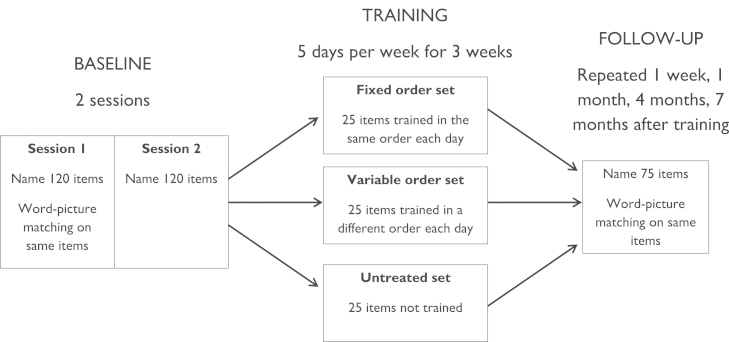
Design of Study 1.

The design of the study is summarised in Fig. 2. Patients first completed a baseline assessment of naming ability in which they named the same set of 120 pictures on two separate occasions. On one occasion, they also performed word–picture matching on the same items, as a measure of comprehension. These results were used to create individual item sets for each patient. For each patient, we generated three sets of 25 items which they had failed to name on both occasions. Match software (van Casteren and Davis, 2007) was used to equate the items in the three sets for the following variables:

1. Patient's accuracy for the items in word–picture matching.
2. Lexical frequency in the CELEX database (Baayen et al., 1993).
3. Rated typicality, familiarity and age of acquisition, all obtained from the norms of Morrow and Duffy (2005).

Each set was assigned to one of three conditions: fixed order, variable order or untreated. Patients then completed three weeks of relearning with the fixed order and variable order items, with the fixed order items being presented to the patients in the same sequence each day, while the variable order items appeared in different sequences. The untreated items were not presented during relearning. Following the training, patients completed follow-up tests after one week and after approximately one month, four months and seven months. In each follow-up session, patients attempted to name all the items from the three sets and performed word–picture matching for these items.

### 2.3 Stimuli

All pictures were black-and-white line drawings obtained from the International Picture Naming Project (Szekely et al., 2004). During naming tests, each picture was presented individually to the patient. For word–picture matching, we constructed a four-alternative test in which each picture was presented in an array with three other pictures from the same semantic category. The name of the item was printed below the array and read aloud by the examiner and the patient was asked to point to the correct picture.

### 2.4 Procedure

The relearning training proceeded as follows. Each patient was given a folder containing the therapy materials. On the first page was a drawing of an object, which they attempted to name. After making an attempt or indicating that they did not know the name, they were asked to turn the page, where the same object was pictured with its name printed below. The patient was asked to read the name aloud and repeat it three times (with any reading errors corrected by the investigator). Training occurred on every day from Monday to Friday for a period of three weeks and took around 20 min each day. The fixed order and variable order items were trained concurrently. Each day's training consisted of a block of the 25 fixed order items and a block of the 25 variable order items, with the order of the two blocks alternating across days. For the fixed order set, we generated one presentation sequence for the items and presented them in this sequence every day. For the variable order items, we constructed five different presentation sequences by randomly shuffling the items. The patient was exposed to a different sequence each day (i.e., each sequence was used once a week).

Therapy of this kind is often administered as homework, with materials left for the patient to work through in their own time. This is practically desirable because it reduces the number of home visits made to the patient, but it can be difficult to monitor and control how much time patients are spending on therapy. In this study, we adopted a different technique in which baseline and follow-up assessments were completed at the patients' homes but the majority of the relearning training was conducted over the telephone. A previous study has shown that this is a suitable method for administering relearning in SD patients (Mayberry et al., 2011a). The advantage of this method from a research perspective is that it eliminates the need for daily home visits but it is still possible to confirm that the patient is engaging in the therapy each day. In addition, performance can be recorded and trends in learning monitored, and any difficulties experienced by the patient can be discussed immediately.

For the follow-up tests, both the fixed order and variable order sets were presented in new sequences that were not used during training. This allowed us to test the degree to which the knowledge acquired for each set of items generalised when they were encountered in an unfamiliar sequence.

### 2.5 Results

#### 2.5.1 Naming performance for trained items

**Fig. 3:**
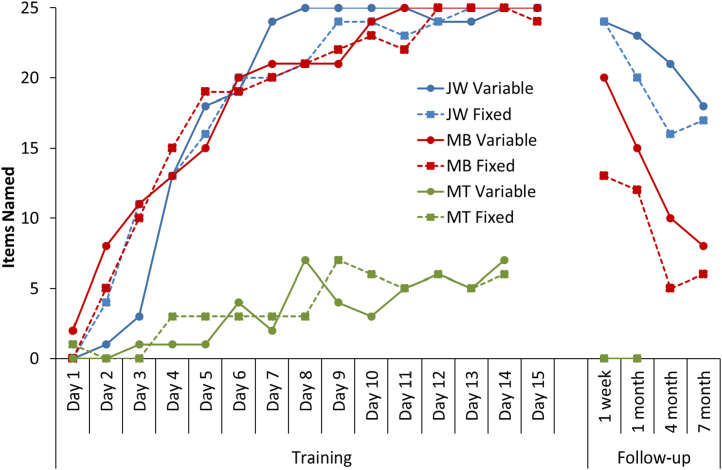
Number of items named correctly during training and follow-up for Study 1. Note: Patient MT did not complete Day 15 of the training, nor the 4 month or 7 month follow-ups.

Naming performance for each patient is shown in Fig. 3. Both MB and JW rapidly learned to name the trained items, reliably naming almost all of the items by the beginning of the third week. In contrast, MT, the most severely impaired patient, struggled to master the training sets and could only name around a quarter of the items by the end of the learning phase. These gains were not maintained at 1-week or 1-month follow-ups, with MT failing to name any items (for this reason, the 4-month and 7-month follow-ups were not completed in this patient). Patients MB and JW, on the other hand, demonstrated much better retention of the trained items, albeit with declining performance over time. During training, both patients demonstrated similar learning curves for the fixed order and variable order sets. However, clear differences emerged in the follow-up sessions, with both patients demonstrating more successful naming of the items that were trained in a variety of different orders. This result indicates that increasing the variability of the learning experience resulted in more durable word learning. The difference between the fixed order and variable order items was statistically significant in patient MB (*χ*^2^=5.85, *p*<0.016, collapsing across all follow-up sessions) though it failed to reach significance for JW (*χ*^2^=2.86, two-tailed *p*=0.1).

#### 2.5.2 Generalisation to untreated items

As expected, the intervention did not improve naming for untreated items. Collapsing across the follow-up sessions, MB correctly named 0/100 untreated items, JW named 1/100 items and MT named 0/50 (note that there were 25 untreated items for each patient, but patients had more than one opportunity to name each one as they completed multiple follow-up sessions).

#### 2.5.3 Errors

Errors made during naming at each stage of the study were analysed to determine if these were affected by the intervention. Naming errors were classified in the following way:

1. Omission: Patient said “don't know” or made no attempt to name.
2. Other trained item: Patient responded with the name of another item from the training set. Occasionally these confusions appeared to be a result of visual similarity (e.g., spider→“octopus”). Typically, however, they were items that belonged to the same semantic category as the target and often did not share much visual overlap (e.g., celery→“artichoke”; lizard→“gorilla”).
3. Semantic co-ordinate error: items from the same semantic category as the target, but which were not part of the training set (e.g., tractor→“car”).
4. Superordinate: Responses that specified the superordinate category to which an item belonged (e.g., donkey→“animal”).
5. Description: Cases where the patient gave some information about the target but did not produce a specific name (e.g., canoe→“you sit in it in the sea”).
6. Phonological error: Response was phonologically similar to the target (e.g., gorilla→“corilla”). Sometimes these responses consisted of a blend of the target name and another trained word (e.g., “twizard” from tweezers and lizard).
7. Other: Responses that bore no clear semantic or phonological relationship with the target. These errors were only common for patient MT, who often gave stock responses (“it's for cleaning” or “it's for playing music”) to items she did not recognise.

**Fig. 4:**
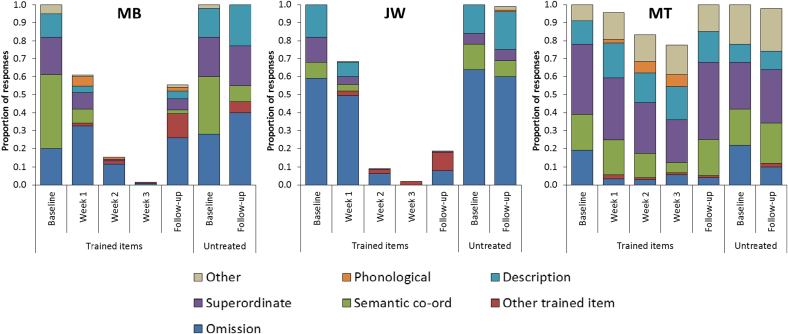
Error types in Study 1. (For interpretation of the references to colour in this figure, the reader is referred to the web version of this article.)

The proportion of errors made by each patient across the key stages of Study 1 are outlined in Fig. 4. Error distributions for fixed order and variable order items were similar so these have been combined in the figure. Omissions are the most common type of naming error in SD generally (Woollams et al., 2008) and this was the case here for patients JW and MB. Semantic co-ordinate errors, superordinate errors and descriptions accounted for the majority of the remainder of errors, highlighting the dissolution of semantic knowledge as the root cause of naming impairment in these patients. The most important feature of these data, however, are the rates at which patients produced the name of another item from the training set (shown in red). Patients MB and JW, who demonstrated the most successful learning, both became susceptible to making these errors following training. This suggests that patients sometimes over-extended names they had learned to other, semantically related concepts. We investigate this possibility in more detail in Study 2. Interestingly, however, both patients were much more likely to make these errors in response to trained items than untreated items. In other words, trained words were not used indiscriminately in response to any stimuli; instead patients were able to restrict their use to items that they had encountered during training.

#### 2.5.4 Word–picture matching

Table 2 shows the proportion of correct responses in the word–picture matching task for each set of items. Patient MT displayed the poorest level of item comprehension at baseline and did not improve at follow-up. MB and JW, however, showed significant improvements on the trained items following the relearning intervention. This indicates that the knowledge gained during naming therapy successfully generalised when the stimuli were encountered in a novel task. There were also slight (non-significant) improvements on the untreated items. This most likely stems from the fact that the word–picture matching trials for untreated items sometimes used trained items as foils and increased knowledge for these might have improved performance. There were no significant differences in word–picture matching performance for fixed order vs. variable order items.

### 2.6 Discussion

The principal finding of Study 1 was that patients demonstrated improved retention of relearnt vocabulary when the training items were presented in a variety of different orders during training. This form of training presumably discouraged the patients from learning the items in “rote fashion”, instead promoting formation of a more flexible item representation that was more easily accessed when the picture was presented at follow-up. The knowledge acquired in the relearning task also generalised to a word–picture matching task that was not trained explicitly. More generally, we observed clear differences between the three patients. MT, the most severely impaired patient, struggled to learn the names during training and demonstrated no retention at follow-up. The other two patients were much more successful and were able to name all of the items by the end of the training phase. Despite similar learning trajectories, JW displayed markedly better performance than MB in the follow-up sessions. We consider these individual differences further in the General Discussion. Finally, we found that MB and JW sometimes incorrectly applied learned names to other semantically or visually related items in the training set. These errors suggest that the patients had difficulty determining exactly which objects the relearned names should be applied to. We investigated this important element of relearning in Study 2.

## 3 Study 2: effect of training with multiple exemplars of each object

In Study 1, we introduced variability into the patients’ learning environment by changing the order in which items were presented each day. Including this variability improved knowledge retention at follow-up. In Study 2, we increased variability by presenting the patients with a number of different pictured examples of each target item. We were particularly interested in the effect that this approach would have on the patients' ability to generalise learning to novel exemplars of the target items. The goal of relearning therapy is to provide patients with vocabulary that they can apply in their everyday lives. To achieve this, use of the learned words must generalise from the specific materials used in therapy to new exemplars of the trained item, even if these are visually distinct. Previous studies indicate that this form of generalisation is limited in SD patients (Heredia et al., 2009; Mayberry et al., 2011a). In particular, Mayberry et al. (2011a) tested generalisation in two patients with SD who were trained to name a single picture for each target word. Following training, patients were asked to name a new example of each trained item, which was visually distinct from that used during training (e.g., patients were trained with a picture of an old-fashioned stove-top kettle and subsequently asked to name a modern electric kettle). Both patients demonstrated poor generalisation to these novel objects, suggesting that the usefulness of the intervention for everyday life may have been limited. Here, we investigated whether providing experience with multiple different exemplars during training would encourage this form of generalisation.

Mayberry et al. (2011a) also explored potential negative effects of generalisation. In their follow-up assessments, they included foils that were visually and semantically related to target items but had different names (e.g., the foil for kettle was a teapot). Following training, both patients began to incorrectly use learned words to name these items. The authors referred to these errors as “over-generalisations”. They suggested that patients had difficulty determining the appropriate conceptual boundaries over which the names should be applied (see also Lambon Ralph et al., 2010). Here, we also included foils in follow-up assessments to investigate the danger that using multiple exemplars during training would encourage this inappropriate form of generalisation.

### 3.1 Participants

MB and JW participated in Study 2. We decided that participation was unlikely to be beneficial for MT, based on her poor performance in Study 1.

### 3.2 Design

**Fig. 5:**
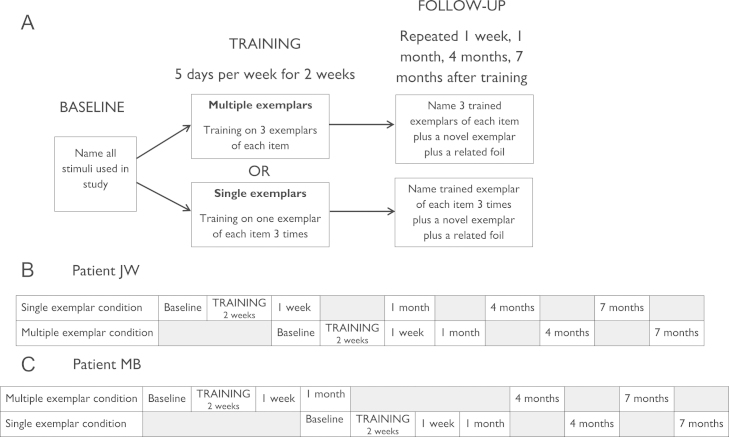
Design of Study 2 and timelines for each patient.

**Fig. 6:**
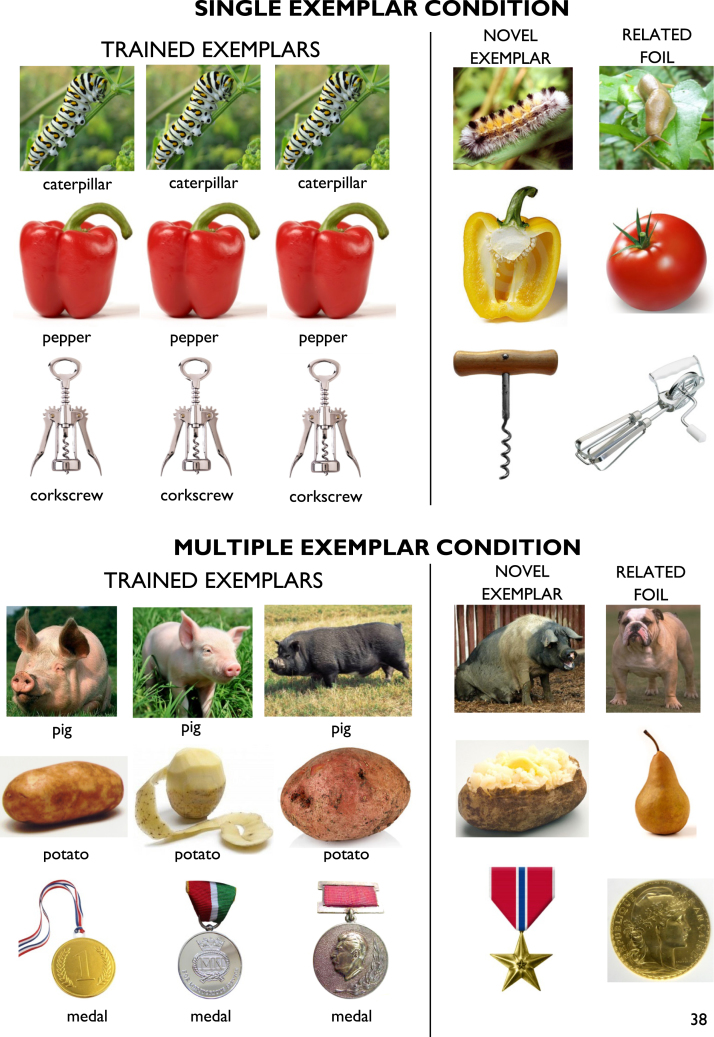
Examples of stimuli in Study 2.

Patients completed two blocks of relearning therapy. The design of each block is summarised in Fig. 5A. In the multiple exemplar (ME) condition, they were trained to name 25 items through exposure to pictures of three different exemplars of the objects (see Fig. 6 for examples). In the single exemplar (SE) condition, they were also trained to name 25 items but were only exposed to one exemplar for each item. To match the total amount of training across the two conditions, this single exemplar was presented three times in each session. Patients also completed a baseline assessment and follow-up assessments at 1 week, 1 month, 4 months and 7 months. Importantly, baseline and follow-up sessions included two additional pictures for each item that probed generalisation behaviour. One was a novel exemplar of the item not used during training and the other was a foil that was semantically and visually similar to the trained item.

In Study 1, patients were trained on both sets of items concurrently. In Study 2, however, the training took longer to administer as each item was presented three times. To avoid over-burdening the patients, we split the training into two blocks. We adopted a cross-over design in which patients received one block of therapy in the SE condition and one block in the ME condition. The order of the blocks was different for each patient. The sequence of events for patient JW is shown in Fig. 6B. She completed the SE baseline and training first. On the day that she completed the 1 week follow-up for this condition, she also completed the baseline for the ME condition and the second block of training began. Subsequent follow-ups for both conditions were interleaved. The sequence for MB was similar except that she completed the ME training first (see Fig. 5C).

### 3.3 Stimuli

Stimuli were colour photographs of objects obtained from the internet. Because of the additional complexity in generating stimuli for this study, we did not attempt to create individual item sets tailored to each patient's naming ability. Instead, we created two sets of items for use in both patients (Sets A and B). We wanted to ensure that the two sets would elicit similar levels of performance at baseline. We therefore matched the 25 items in each set for lexical frequency, familiarity and age of acquisition, since these variables strongly influence naming ability in SD. The assignment of item sets to experimental conditions was also counterbalanced across the two patients: JW received the Set A items in the SE condition and the Set B items in the ME condition while the reverse was true for MB.

For each item, we found four photographs of different exemplars. We selected images that varied as much as possible in their perceptual characteristics while still being clearly recognisable as the object in question (see Fig. 6). Three of these images were used in ME training (one image in SE training). The final image was retained for use as a novel exemplar, to test generalisation of learning at follow-up. For each item, we also selected a related foil, which was an object that shared semantic and visual characteristics with the trained item but had a different name. The foil was not used in training but was presented at follow-up to test whether patients would incorrectly over-generalise learned names to related concepts.

### 3.4 Procedure

The method for relearning was similar to Study 1, with patients attempting to name each picture before turning the page to reveal the correct name, which they then repeated three times. Patients saw 75 pictures each day, divided into three sections. In the ME condition, the first section consisted of one picture for each item in the training set. The second section consisted of a picture of a different exemplar for each item, presented in a different sequence. The third section contained another exemplar for each item, again in a different sequence. The SE condition was identical except that the same pictures were presented in each section. Therefore the total amount of practice with each word was equated across conditions. Relearning took place for five days a week over two weeks and was again administered over the telephone.

In the baseline and follow-up assessments for the ME condition, patients named 125 pictures, comprising the three trained exemplars for each item, their novel exemplars and their foils. The order of the 125 pictures was randomised and the patients were told beforehand that some of the pictures would be the ones they practiced, some would be new pictures that had the same names as the practiced ones and some were new pictures that might look similar but had different names. Assessment for the SE condition was the same, except each of the 25 trained exemplars was presented three times. This was to ensure that the patients would have the same number of opportunities to produce the correct name as in the ME condition.

### 3.5 Results

#### 3.5.1 Naming performance for trained items

**Fig. 7:**
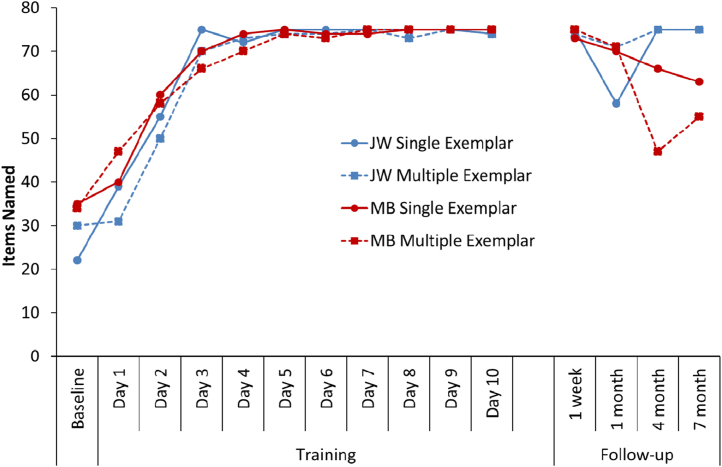
Number of items named correctly during baseline, training and follow-up for Study 2.

Each patient's naming performance during training and at follow-up is shown in Fig. 7. Unlike Study 1, we did not specifically select items that the patients were unable to name. As a consequence, both patients were able to name between one-third and a half of the items at baseline. At baseline, MB named an equivalent number of SE and ME items, but JW's baseline was slightly better for ME items. With training, performance rapidly increased, reaching a plateau by the end of the first week, and with no obvious differences between conditions. Learning was maintained very well at follow-up, though there were two anomalous data-points worth mentioning. For JW, there was a pronounced drop for the SE items at the 1 month follow-up. This is unsurprising because between the 1 week and 1 month follow-ups for these items, JW completed the training for the ME items (see Fig. 5B). The drop in performance may therefore indicate interference caused by learning a new set of items. Importantly, this was a temporary effect: naming returned to 100% accuracy at later follow-ups. A similar temporary interference effect can be seen for MB, though in her case this occurred at the 4 month follow-up for the ME items (cf. Fig. 5C). If we ignore these temporary blips in performance, there was little difference between SE and ME naming at follow-up. Chi-square tests (collapsing across follow-ups but excluding the anomalous time points) revealed no difference between conditions for either patient (JW: *χ*^2^=1, *p*=0.32; MB: *χ*^2^=0.64, *p*=0.42).

#### 3.5.2 Generalisation to novel exemplars

**Fig. 8:**
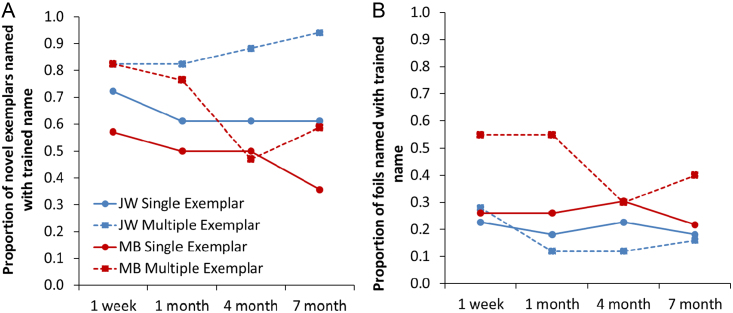
Production of trained names in response to novel exemplars and foils in Study 2.

We next examined how successfully patients were able to apply learned names to new exemplars of the same object. Naming for the novel exemplars was tested at baseline and at each follow-up point. It was important to test these stimuli at baseline so that we could distinguish training-induced gains in naming from cases where the patient was able to name the item prior to the study. To focus on improvements caused by training, we excluded from the analysis any novel exemplars that the patients named correctly at baseline. The percentage of novel exemplars named correctly at each follow-up session is presented in Fig. 8A. Both patients displayed more successful generalisation for item names that were trained using multiple exemplars. This effect was significant for patient JW (*χ*^2^=9.76, *p*=0.002) and for MB (*χ*^2^=4.07, *p*=0.04). The effect was apparent at every follow-up point with the exception of MB's 4 month follow-up, where ME knowledge was temporarily affected by recent learning of the SE items (as explained earlier).

#### 3.5.3 Over-generalisation to foils

Finally, we investigated the possible negative consequence of increased generalisation: that patients would apply the learned names incorrectly to foils that shared semantic and visual characteristics with the trained items. We tested naming for the foils at baseline and found that there were some foils for which the patients already erroneously used the name that was going to be the therapy target. We excluded these from the analysis, as it was not possible for the patients to develop over-generalisation to them as a consequence of training. Fig. 8B shows the rates of over-generalisation for the remaining foils. JW was largely successful in avoiding over-generalisation errors: typically she only used the trained name on around 10–20% of trials. MB was less successful, particularly for foils in the ME condition. A chi-square test indicated that MB was more likely to over-generalise trained names in the ME condition, compared with the SE condition (*χ*^2^=6.74, *p*=0.009). There was no such effect in JW's performance (*χ*^2^=0.37, *p*=0.54). In MB’s case, therefore, there was evidence that training with multiple exemplars had a detrimental effect in terms of increasing over-generalisations. Most worryingly, there were five foils (3 SE, 2 ME) that MB named correctly at baseline which she switched to naming with the trained words at follow-up. Therefore, her gains in naming the trained items came at the cost of reductions in correct naming for semantically related items. JW did not experience such problems. Table 3 shows *correct* naming of foils at each stage of the study. There was a tendency for JW's naming of the foils to improve during the course of the study, suggesting that training on their semantic neighbours may have benefited the foils in her case.

### 3.6 Discussion

The principal finding of Study 2 was that exposure to multiple exemplars of each item during relearning improved the patients' ability to generalise learned vocabulary to new exemplars. In MB's case, however, these gains came at a cost: she became more likely to erroneously use the learned words to name other objects that were visually and semantically similar to those used in training. In some cases, these over-generalisations even appeared to “overwrite” knowledge that MB had prior to the intervention (though this particular problem was not limited to items trained with multiple exemplars). In contrast, the use of multiple exemplars improved JW’s ability to generalise item names appropriately, with no concomitant effects on over-generalisation.

## 4 General discussion

The goal of anomia therapy is to provide patients with vocabulary that they can use in their everyday lives. This means that, for maximum benefit, knowledge about items acquired in the therapy setting must generalise to experience with the same items in novel situations. Previous studies suggest that such generalisation is limited in patients with SD (Mayberry et al., 2011a). We investigated two manipulations designed to promote generalisation of learned vocabulary in these patients. Both manipulations occurred in the context of picture-name relearning interventions. First, we presented items for training in a different sequence each day, to discourage learning of lists in rote fashion, and compared this with training in the same order each day. Patients performed better on items trained in variable orders, when these were tested in a novel order at follow-up. In the second study, we presented patients with three different exemplars of each training item and compared this with an equivalent amount of training with only one exemplar. When words were trained using multiple exemplars, word use generalised more successfully to novel exemplars of the same object. In one patient, however, this positive effect was offset by an increase in over-generalisation errors, in which she inappropriately used the trained labels to name other, semantically related objects. We will now consider theoretical explanations for these results, possible explanations of the individual differences between patients and the implications of these findings for the design of anomia therapies.

### 4.1 Theoretical account

As outlined in the Introduction, popular theories of learning hold that new information is initially coded in a hippocampal learning system and is transferred to neocortical sites through a process of gradual consolidation (Alvarez and Squire, 1994; McClelland et al., 1995). The hippocampal system employs sparse representations that record the details of individual experiences. The consolidation process, on the other hand, extracts statistical regularities over many experiences while discarding the idiosyncratic elements of each individual episode. This results in representations of the *typical* properties of objects and events that are true across many different situations – i.e., semantic knowledge. Importantly, this aggregated knowledge is readily generalisable to new situations (Lambon Ralph et al., 2010). In SD, consolidated semantic representations in the anterior temporal lobes are severely affected (Bozeat et al., 2000; Mion et al., 2010; Patterson et al., 2007). The hippocampal learning system, while not entirely spared, is affected to a much lesser extent (Graham et al., 2000; Hoffman et al., 2014; Nestor et al., 2006). It has been claimed that SD patients rely mainly on the hippocampal system for learning new material and, as a consequence, their representations are highly rigid and context-dependent and do not generalise successfully to novel situations (Graham et al., 1999; Henry et al., 2008; Heredia et al., 2009).

There are two ways of considering the effects of our manipulations within this theoretical framework. The first is that by varying the patients' experience during learning, we improved the usefulness and accessibility of the hippocampal memory traces. For example, in Study 2, the association of the same name with three different images may have encouraged the formation of three different hippocampal representations. When patients then encountered the novel exemplar, if it was sufficiently similar any of one of these representations could trigger retrieval of the name. According to this view, there was nothing inherently “semantic” about the training we provided. Instead, patients may have approached the training as a paired-associate learning task and learned the mappings between pictures and labels without engaging with the potential conceptual significance of the images themselves. The second possibility is that, by varying the experience during learning, we shifted the division of labour away from the hippocampal system and toward the neocortical, semantic system. It could be that the hippocampal learning system was not well-suited to acquiring the mappings between pictures and words in more varying situations and so, to succeed during training, the patients were forced to make use of their remaining semantic resources. Involvement of the semantic system in relearning is supported by evidence that the level of remaining semantic knowledge predicts relearning success in SD patients. This is true at the level of individuals, where patients with milder semantic impairments tend to benefit more from relearning (Graham et al., 1999; Henry et al., 2013; Mayberry, 2011a; though there are also cases with severe semantic impairment who have made substantial gains, e.g., Heredia et al., 2009; Savage et al., 2013), and at the level of items, where object names are more likely to be retained when the patient has some remaining knowledge for the object (Jokel et al., 2010, 2006). These findings suggest that object names are more likely to be retained when they can be grafted onto elements of retained knowledge in the semantic system. The distinctive pattern of under-generalisation to novel exemplars of the same item and over-generalisation to related items observed in the present study also suggests involvement of the semantic system, as breakdown of existing semantic knowledge in SD follows exactly the same pattern (Lambon et al., 2008; Lambon Ralph et al., 2010; Mayberry et al., 2011b). When categorising objects, for example, patients come to rely increasingly on superficial, surface similarities rather than true conceptual significance (Lambon Ralph et al., 2010; Mayberry et al., 2011b). This can cause them to both under-generalise category labels to atypical members of the category while over-generalising to “pseudo-typical” items that share some superficial similarities (e.g., rejecting an ostrich as an example of a bird but accepting a butterfly). It is likely then that relearning in SD does involve the semantic system to some extent and that our manipulations shifted learning from the hippocampal and toward the semantic system. If this were the case, then we would expect the integrity of the semantic system to be an important factor in determining the success of the interventions. We consider this possibility next.

### 4.2 Explaining individual differences

While a consistent story has emerged across the two studies, there were also important individual differences between the three patients. In Study 1, JW and MB both learned all of the items (though JW demonstrated better performance at follow-up), while MT displayed limited learning and no maintenance at follow-up. In Study 2, both patients demonstrated improved generalisation when treated with multiple exemplars but only MB showed a concomitant increase in over-generalisations. In fact, JW represents a notable success story in terms of relearning interventions in SD. In Study 2, she maintained naming ability for all 50 of the trained items, even seven months after the intervention. This is a much higher level of maintenance than seen in most patients. Moreover, when trained with multiple exemplars, she was able to generalise names to around 90% of new exemplars at follow-up while only incorrectly generalising to around 10% of foils. In Study 2, she even showed signs of improvement on untreated foils. What underlies this unusually successful response to therapy? One factor was probably JW’s good level of remaining semantic knowledge for items she could not name. In Study 1, JW's accuracy on word–picture matching exceeded that of the other patients. Similarly, at the start of Study 2, JW and MB had similar baseline naming scores but JW’s general level of comprehension was better (see scores for word–picture matching and synonym judgement in Table 1). Remaining semantic knowledge for therapy items seems to be an important factor in determining response to therapy in this group (Jokel et al., 2010, 2006; Snowden and Neary, 2002). This is presumably because the long-term success of relearning depends on new vocabulary being integrated into the semantic knowledge system, since hippocampal representations decay quickly (Murre et al., 2001). Residual knowledge for trained items is also likely to be critical for ensuring that vocabulary is generalised appropriately, since this requires that the patient maintains a conceptual boundary for the word that allows them to decide which items should be named with it and which should not (Lambon Ralph et al., 2010).

JW's profile of relatively good comprehension for many items she could not name was accompanied by asymmetric anterior temporal lobe atrophy that disproportionately affected the left hemisphere. Patients with this form of atrophy often present with a pronounced anomia but retain some semantic knowledge for items they cannot name (Lambon Ralph et al., 2001). The present study suggests that these patients with this presentation may be especially good candidates for relearning therapy. In contrast, MT, the most severely impaired patient, had more severe atrophy in the right anterior temporal lobe and demonstrated no recognition for many of the items she could not name (e.g., screw→“it’s for making music”). In her case, therefore, there was little remaining knowledge for the names used in training to become associated with. This may explain her limited capacity for relearning.

**Fig. 9:**
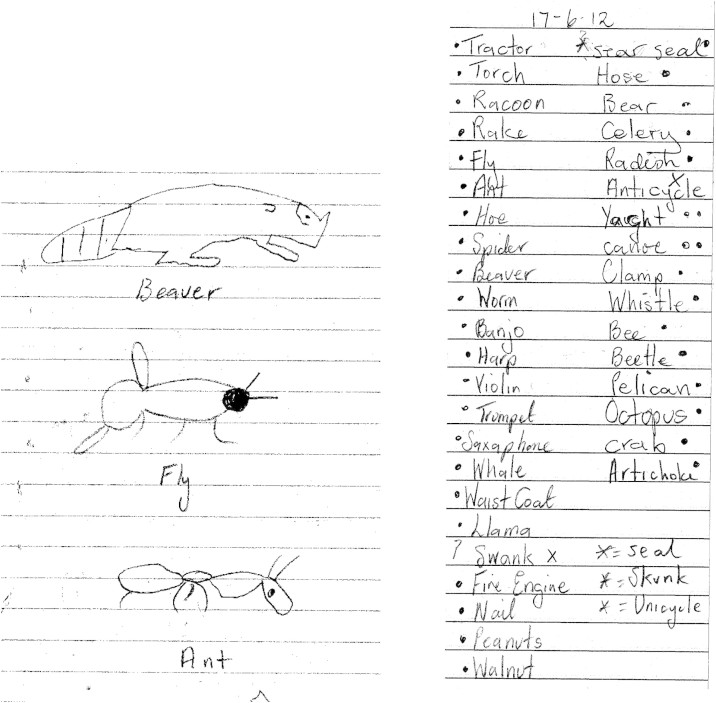
Examples of patient JW's self-initiated practice during relearning.

Another important factor is motivation and willingness to participate in practice. A number of previous studies have noted that SD patients who are strongly motivated to recover vocabulary show good outcomes following therapy (Heredia et al., 2009; Jokel et al., 2006; Snowden and Neary, 2002). One particularly striking example is that of patient DM, who was able to stave off reductions in vocabulary through many hours of self-initiated practice (Graham et al., 1999). In fact, prior to the current study, JW also engaged in self-initiated practice, using lists of words she had forgotten and scrapbooks of pictures she had compiled. She also performed additional practice during the training phases of both studies, which likely boosted her performance. We instructed her to only look at the therapy materials once a day during the telephone session and her husband reported that she complied with this. In addition to this, she did, however, practice by listing the words and drawing the pictures from memory (see Fig. 9 for examples from Study 1). She did not report engaging in this activity for one condition more than the others, so it is unlikely to have influenced the advantage seen for more variable training regimes. Nor did the additional practice appear to increase her rate of initial learning, which was similar to that of MB. It may, however, have contributed to her good retention of the items at follow-up. In contrast, neither MB nor MT reported doing additional practice.

In summary, there are two main factors that might account for JW's exceptional response to relearning therapy: level of remaining knowledge for items she could not name and strong intrinsic motivation to recover lost vocabulary. It is also worth noting that, following Study 1, JW incorporated the relearning method used in the study into her own self-directed practice. She took photographs of objects from around the house on a digital camera and regularly practiced naming these on the camera's screen, with her husband correcting any errors. The set of photos was updated periodically when she reached a certain level of proficiency. She reported finding this a very useful technique. In fact, this approach was likely to be of more functional value to her than the relearning we administered formally, as it used personally relevant objects which typically result in more successful relearning (Jokel et al., 2006; Savage et al., 2013; Snowden and Neary, 2002).

### 4.3 Implications for anomia therapy

The results of the present study have a number of implications for design of anomia therapies in SD. The outcome of Study 1 was clear-cut: both patients who were able to learn the training sets showed better retention for items that were trained in a variety of different orders. While some other relearning studies have adopted variable order presentation to discourage rote learning (e.g., Savage et al., 2013), to our knowledge this is the first study to compare this technique directly with the more usual fixed order presentation. We suggest that future studies consider varying item order to maximise therapy gains. A more complex picture emerged from Study 2. Both patients benefited from training with multiple exemplars of each item, in the sense that this improved generalisation to new, perceptually distinct exemplars of the items used in training. In MB's case, however, it also increased inappropriate over-generalisations of the trained name to semantically related concepts. This is a potential detrimental consequence of attempts to improve generalisation, which should be explored in more detail to determine under what circumstances it is likely to occur. JW was not vulnerable to over-generalisation and we have speculated that this was due to her level of preserved knowledge for the items used in training. Clearly, this finding requires replication in other patients. At the very least, clinicians should be aware of the possible negative consequences of promoting generalisation in this group. As Mayberry et al. (2011a) noted, such over-generalisations may go unnoticed if the therapy materials are not designed to detect them (i.e., by probing items with a close semantic or visual relationship to the trained targets).

Poor generalisation has often been noted as a problem in the relearning of SD patients. JW's experience suggests that appropriate generalisation is possible, at least for patients with relatively mild comprehension impairments. More generally, the success of this patient highlights the utility of interventions that engage the semantic system rather than relying on hippocampal rote learning. Other researchers have done this in a much more direct fashion than we have here, by incorporating meaning-based tasks such as feature generation (Bier et al., 2009; Henry et al., 2013) or using meaningful descriptions as additional cues for naming (Savage et al., 2013; Snowden and Neary, 2002). While this engagement of the semantic system in some patients is encouraging, its success depends on the presence of residual semantic knowledge to which vocabulary can attach itself. In particular, the ability to generalise labels appropriately, which is one of the key functions of semantic knowledge, is likely to diminish as the disease progresses.

## Figures and Tables

**Table 1:** Background neuropsychological data.

Test	Max	MB	MB	JW	JW	MT	Control mean (minimum)
Jan 2012	March 2013	May 2012	Feb 2013	Sept 2012
*General cognition*							
MMSE	30	26	27	28	28	**22**	28.8 (24)
ACE-R	100	**64**	**69**	**69**	**60**	**51**	93.7 (85)
Ravens coloured progressive matrices	36	33	32	31	NT	33	
Forward digit span	–	6	5	6	NT	8	6.8 (4)
Backward digit span	–	4	4	6	NT	7	4.8 (3)
Letter fluency (FAS)	–	18	**14**	18	27	**15**	41.1 (17)
							
*Visuospatial*							
Rey copy	36	34	**30**	34	NT	36	34.0 (31)
VOSP incomplete letters	20	20	20	20	20	**15**	18.8 (16)
VOSP number location	10	10	10	10	9	10	9.4 (7)
							
*Semantic cognition*							
Category fluency	–	**33**	**26**	**24**	**17**	**15**	95.7 (62)
Picture naming	64	**19**	**20**	**26**	**18**	**11**	62.3 (59)
Word–picture matching	64	**36**	**40**	**52**	**49**	**16**	63.8 (62)
Camel & Cactus Test	64	**38**	**36**	**42**	NT	NT	59.1 (51)
Synonym judgement	96	**69**	**63**	**80**	**75**	**68**	94.5 (91)

Control data were obtained from published norms. Minimum control score indicates cut-off below which performance is considered abnormal (two standard deviations below the mean if no other threshold was provided). MMSE=Mini-Mental State Examination (Folstein et al., 1975), ACE-R=Addenbrooke's Cognitive Examination (Revised) (Mioshi et al., 2006), VOSP=Visual Object and Space Perception Battery (Warrington and James, 1991), and NT=not tested. Bold values indicate abnormal scores.

**Table 2:** Word–picture matching performance in Study 1.

Patient	Set	Baseline (%)	Follow-up (%)			
1 week	1 month	4 months	7 months
MB	Fixed order	48	NT	**84**	**72**	64
Variable order	48	NT	**88**	**80**	**76**
Untreated	48	NT	68	52	44
						
JW	Fixed order	60	**92**	**100**	**100**	84
Variable order	60	**100**	**96**	**88**	**92**
Untreated	60	80	76	60	76
						
MT	Fixed order	28	24	20	NT	NT
Variable order	28	36	24	NT	NT
Untreated	28	32	28	NT	NT

Follow-up performances that are significantly improved, relative to baseline, are shown in bold (McNemar’s test; one-tailed *p*<0.05). Due to an administrative error, MB did not complete word–picture matching in the 1 week follow-up session.

**Table 3:** Accurate naming of foils in Study 2.

Patient	Set	Baseline (%)	Follow-up (%)			
1 week	1 month	4 months	7 months
MB	Single exemplar	28	24	16	24	16
Multiple exemplars	20	20	16	12	16
						
JW	Single exemplar	20	24	16	**44**	**44**
Multiple exemplars	28	24	28	40	40

Follow-up performances that are significantly improved, relative to baseline, are shown in bold (McNemar's test; one-tailed *p*<0.05).
